# Highly Efficient Methods to Culture Mouse Cholangiocytes and Small Intestine Organoids

**DOI:** 10.3389/fmicb.2022.907901

**Published:** 2022-05-20

**Authors:** Wenyi Chen, Qigu Yao, Ruo Wang, Bing Fen, Junyao Chen, Yanping Xu, Jiong Yu, Lanjuan Li, Hongcui Cao

**Affiliations:** ^1^State Key Laboratory for the Diagnosis and Treatment of Infectious Diseases, Collaborative Innovation Center for Diagnosis and Treatment of Infectious Diseases, The First Affiliated Hospital, Zhejiang University School of Medicine, Hangzhou, China; ^2^National Clinical Research Center for Infectious Diseases, Hangzhou, China; ^3^Zhejiang Provincial Key Laboratory for Diagnosis and Treatment of Aging and Physic-chemical Injury Diseases, Hangzhou, China

**Keywords:** organoids, cholangiocytes, small intestine, 3D culture, intestinal crypt, liver

## Abstract

**Background:**

Organoids, which enable disease modeling and drug screening closer to an *in vivo* environment, can be isolated and grown from organs such as the brain, small intestine, kidney, lungs, and liver. To facilitate the establishment of liver and small intestinal organoids, we developed efficient protocols for cholangiocytes and intestine crypts collecting and organoid culturing.

**Methods:**

Cholangiocytes were collected from intrahepatic bile ducts, the gallbladder, and small intestine crypts by gravity settling and multistep centrifugation methods. The cells isolated were embedded with Matrigel and grew in three-dimensional spheroids in a suitable culture medium. The stability of organoid cells was assessed by subculture, cryopreservation, and thawing. RNA and DNA extraction of organoids, as well as immunostaining procedure, were also optimized. Hand-picking procedures were developed and performed to ensure similar growth characteristics of organoids.

**Results:**

A large number of cholangiocytes and small intestine crypts were collected under these protocols. Cholangiocytes developed into cyst-like structures after 3–4 days in Matrigel. After 1–2 weeks of cultivation, small intestinal organoids (in-orgs) developed buds and formed a mature structure. Compared to organoids derived from the gallbladder, cholangiocyte organoids (Cho-orgs) from intrahepatic the bile ducts grew more slowly but had a longer culture term, expressed the cholangiocytes markers Krt19 and Krt7, and recapitulated *in vivo* tissue organization.

**Conclusions:**

Our protocols simplified the cell collection procedure and avoided the possibility of exposing tissue-derived stem cells to mechanical damage or chemical injury by gravity settling and multistep centrifugation. In addition, our approach allowed similar growth characteristics of organoids from different mammalian tissue sources. The protocol requires 2–4 weeks to establish a stable organoid growth system. Organoids could be stably passaged, cryopreserved, and recovered under protocol guidance. Besides, the organoids of cholangiocytes and small intestines retained their original tissue characteristics, such as tissue-specific marker expression, which prepares them for further experiments such as preclinical *in vitro* trials and mechanism research studies.

## Introduction

Organoids are *in vitro* three-dimensional (3D) cultures grown from primarily isolated cells or stem cells (Dutta et al., 2017). Three-dimensional culture enables organoid growth from healthy (Huch and Koo, 2015) or diseased (van de Wetering et al., 2015) human or animal primary tissues. Organoids have potential as preclinical models (Lancaster and Knoblich, 2014; Dutta et al., 2017; Zhang et al., 2021) for drug screening (Kondo and Inoue, 2019) and in mechanistic research (Lancaster and Knoblich, 2014; Vives and Batlle-Morera, 2020). Standardized good manufacturing practice (GMP)-compliant scalable organoids may enable the replacement of damaged human organs (Dossena et al., 2020). What's more, in the widespread spread of world pandemics such as SARS-CoV-2, organoids were also applied to evaluate the intestinal function changes (Zhou et al., 2020), which disclosed a deeper insight into unique gene mutations in gastrointestinal disease (Li, 2021).

As for the cellular composition of organs, the liver mainly comprises hepatocytes, cholangiocytes, and nonepithelial cells (Duncan et al., 2009). The small intestine is almost formed of the intestinal epithelium, which contains intestinal crypt-villus units and Lgr5^+^ stem cells (Sato et al., 2009), which potentially own stem cell properties. For organoid growth, Matrigel is needed to provide an *in vitro* 3D environment for stem cell growth. Besides, the proliferation of hepatic and small intestinal stem cells requires epidermal growth factor (EFG), the R-spondin 1 (R-SPO1) protein (notch signaling components) (Sato et al., 2009), growth factors (e.g., N2, B27, and Noggin), hepatocyte growth factor (HGF), and fibroblast growth factor (FGF), as mentioned before.

**Figure 1:**
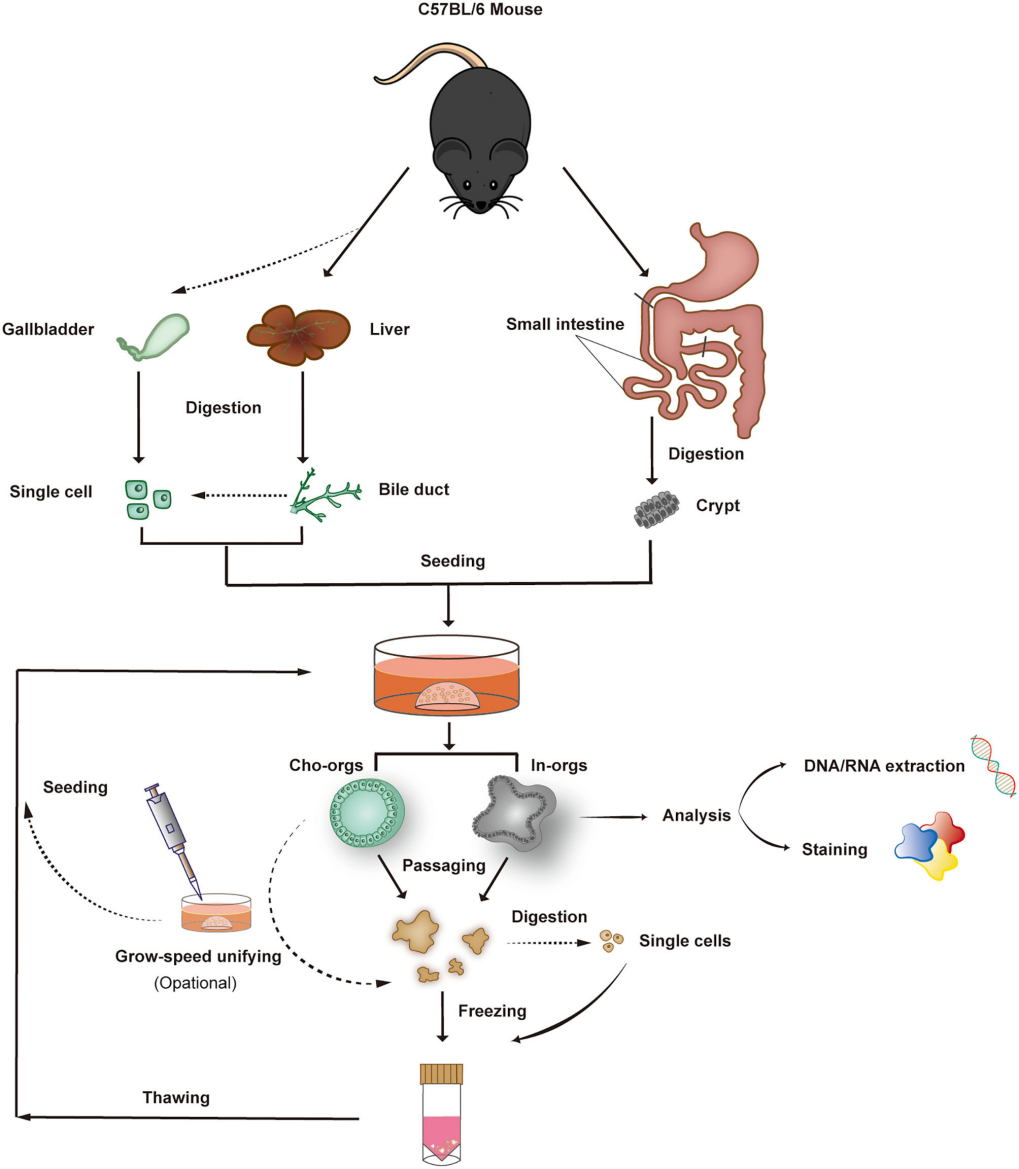
Organoid isolation, culture, growth-rate harmonization, passaging, and freezing. Bile ducts, single bile duct (or gallbladder) cells, and crypts from the small intestine derived from C57BL/6 mice form 3D structures after 7–14 days of culture (5–7 days for gallbladder-derived organoids) in Matrigel. After expansion, the organoids can be characterized or frozen in liquid nitrogen.

Based on the above research studies, we developed and improved an efficient method for collection of cholangiocytes and intestinal crypts by gravity settling and multistep centrifugation, which not only simplified the steps of cell separation but also improved the rate of cell acquisition. However, during the experiment, we found a phenomenon that stem cells derived from different mice present different growth characteristics, with some developing into mature organoids in 7–10 days and the others taking more than 10 to develop into mature forms. Therefore, we established an organoid hand-picking procedure (Figure 1) to ensure that the organoids had similar growth characteristics and avoid excessive differences in organoid growth, which is convenient for disease modeling and mechanism research.

Under our protocol guidance, the organoids can be passaged, cryopreserved, and thawed, and they are suitable for genetic analysis and immunochemical staining. Compare with other stem cell isolation methods, our procedure does not require additional mechanical equipment (e.g., flow cell sorter) and chemical experiments, like antibody combination, which avoids the possibility of exposing stem cells to mechanical damage or chemical injury. Besides, our protocol makes it is easy for beginners to get started and does not require physical exertion. Most importantly, the cell growth rates of stem cells gained from different mammals are unified before further experiments.

Organoids generated from the bile ducts and small intestine have the characteristics of the original tissue, which makes them suitable for disease modeling, drug screening, mechanism research, etc. As recent studies show, organoids may also have a therapeutic investigation potential for diseases such as primary sclerosing cholangitis (PSC) (Loarca et al., 2017), intestinal cancer (Aberle et al., 2018), and inflammatory bowel diseases (IBD) (Meir et al., 2020). Our protocol may provide huge support for the early-stage preparation of such research studies.

## Experimental Design

### Cell Isolation

To isolate bile ducts (cholangiocytes) from mouse liver, the ducts and gallbladder can be digested. In brief, the liver was perfused with PBS 1× through the inferior vena cava before removing the liver tissue. If a small number of cholangiocytes is needed, or if cho-orgs must be cultured rapidly, cholangiocytes can be obtained from mouse gallbladder. After digestion, ducts and crypts from the small intestine can be collected by gravity settling followed by multistep centrifugation. If cholangiocytes need to be counted, bile ducts can be further digested using a single-cell TrypLE Express medium.

### Organoid Culture and Establishment of Stable Organoid Lines

To establish a long-term organoid culture, we picked and collected larger organoids to passage and to unify the whole growth speed of organoids in the same culture well. As reported by Huch, when culturing organoids for further experiment, the elements of organoid size, proliferation rate, physical morphology, formation rate, and expression of markers/genes are all needed to be considered (Huch et al., 2013). Therefore, we described an easy hand-picking procedure to achieve this goal. We also provided two methods of cho-orgs culture, one from intrahepatic bile ducts and the other from gallbladder cholangiocytes. These two types of organoids both expressed cholangiocyte specific markers like Krt7 and Krt19, and displayed different transcriptional profiles and differentiation capacity as demonstrated by Casey A. Rimland (Rimland et al., 2021), which provided a basis for selection according to purpose, such as disease simulation or *in vitro* modeling. Besides, the methods of home-made Wnat3a and R-SPO1 were described by Broutier (Broutier et al., 2016), which were indispensable in organoid culture. When a stable culture line is established, organoids can be maintained for a long time.

### Organoid Analysis

To research the gene changes of the organoids, after the removal of Matrigel from the organoid cultures, the procedure of DNA or RNA extraction from the organoids was almost similar with normal cells. Matrigel was removed first using a pre-cold basal medium. It was suggested to use a cell recovery solution to remove the Matrigel without disrupting organoid structures for immunofluorescence and immunohistochemical staining analysis. Whole-mount immunofluorescence staining enabled 3D structure observation and characterization, such as detecting aimed antibody signal distribution. For observation of cellular morphology and storage of organoids for further staining analysis, paraffin sectioning and immunohistochemical staining are preferred.

## Materials

### Animals

Male or female mice of any genetic background and ranging in age from 6 weeks to 1.5 years (weight 20–28 g) can be used for cho-org culture; for in-org culture, ≤ 6-week-old C57BL/6 mice should be used.

### Reagents

- Collagenase D (Roche, cat. no. 1108866001).
- Dispase II (Life Technologies, cat. no. 17105-041).
- DNase I (Sigma-Aldrich, cat. no. DN25).
- TrypLE Express (Life Technologies, cat. no. 12605-028).
- EDTA (Sangon Biotech Inc., Shanghai, CAS: 60-00-4).
- Matrigel matrix, phenol-red-free (BD, cat. no. 356231).
- Advanced DMEM/F-12 (Life Technologies, cat. no. 12634-010).
- DMEM, high glucose, GlutaMAX, pyruvate (Life Technologies, cat. no. 11995-065).
- GlutaMAX (100×; Life Technologies, cat. no. 35050-068).
- HEPES (Life Science Products & Services, cat.no. HB0264).
- Penicillin/streptomycin (Corning, cat.no.30002297).
- B27 Supplement 50×, minus vitamin A (Life Technologies, cat. no. 12587-010).
- N2 Supplement 100× (Gibco, Life Technologies, cat. no. 17502-048).
- L-glutamine (Gibco, Life Technologies, cat. no.25-005-CI).
- N-acetylcysteine (Sigma-Aldrich, cat. no. A0737-5MG).
- Nicotinamide (Sigma-Aldrich, cat. no. N0636).
- Recombinant mouse (Rm) FGF10 (Peprotech, cat. no. 100-26).
- Rm EGF (Life Technologies, cat. no. PMG8043).
- Rm HGF (Peprotech, cat. no. 100-39).
- [Leu15] - Gastrin I human (PL Laboratories, cat. no. P2000646).
- Rho kinase inhibitor Y-27632 dihydrochloride (Sigma-Aldrich, cat. no. Y0503).
- Recombinant human (Rh) Noggin (Peprotech, cat. no. 120-10C).
- Wnt3a-conditioned medium (home-made).
- Rspo1-conditioned medium (home-made).
- Freezing solution (Life Technologies, cat. no. 12648-010).
- Cell recovery solution (Corning, cat. no. 354253).

Additional reagents (Supplementary Table S1), instruments (Supplementary Table S2), and antibodies (Supplementary Table S3) are listed in Supplementary Materials.

## Reagent Setup

### Mouse Liver Digestion Medium

The digestive medium should be prepared fresh and used immediately. Collagenases D and II were dissolved in a sterile washing medium (see below) at a concentration of 0.125 mg/ml and supplemented with 0.1 mg /ml DNase I (dissolved in sterile H_2_O).

### Mouse Liver Basal Medium

Advanced DMEM/F-12 was added with 1% penicillin/streptomycin, 1% GlutaMAX, and 10 mM HEPES. It can be stored at 4°C for 1 month.

### Mouse Liver Isolation Medium

The mouse liver isolation medium was a mouse liver expansion medium supplemented with 25 ng/ml recombinant human Noggin, 30% (vol/vol) Wnt3a-conditioned medium, and 10 μM Rho kinase (ROCK) inhibitor (Y-27632 if single cells were cultured). The medium was stored at 4°C for up to 2 weeks.

### Mouse Liver Wash Medium

The DMEM (high glucose, GlutaMAX, and pyruvate) supplemented with 1% FBS and 1% penicillin/streptomycin. The medium was stored at 4°C for up to 1 month.

### Mouse Liver Expansion Medium

Mouse liver basal medium supplemented with B27 50×, 1 mM N-acetylcysteine, 5% (vol/vol) Rspo1, 10 mM nicotinamide,10 nM Gastrin I, 50 ng/mL EGF, 100 ng/ml FGF10, and 50 ng/ml HGF. Store the medium at 4°C for up to 2 weeks.

### Mouse Intestine Expansion Medium

Advanced DMEM/F-12 supplemented with 10 mM HEPES, 2 mM L-glutamine, N2 50×, B27×, 50 ng/ml EGF, 100 ng/ml Noggin, and 10% (vol/vol) R-spondin 1. Store it at 4°C for up to 1 month.

### Mouse Intestine Digestion Medium

The PBS 1× supplemented with 10 mM HEPES, 1%(vol/vol) L-glutamine, 1 mM EDTA, 1% penicillin/ streptomycin, and 5% FBS.

### Regent Preparation for Immunofluorescence (IF)

PBS-BSA 1% (vol /vol): 1 g BSA per 100 ml PBS 1×. Store at 4°C for 2 weeks.

PBT 0.1% (vol /vol): 1 ml Tween 20 per 1,000 ml PBS 1×. Store at 4°C for 4 weeks.

Washing buffer: 1 ml Triton X-100 and 2 g BSA per 1 L PBS 1×. Store at 4°C for 2 weeks.

F-G clearing solution: 2.5 M fructose and 60% glycerin. Store at 4°C in the dark for up to 1 month.

### Regent Preparation for Immunohistochemistry (IHC)

Alcohol 96% (vol/vol): dilute 100% alcohol with purified water.

Eosin solution 0.5% (wt/vol): Dissolve 0.5 g eosin in 100-ml 96% alcohol.

## Procedure

I. Culture of mouse cholangiocytes and small intestinal organoids: cell isolation
Mouse liver duct cells may be collected from the liver or gallbladder.

**Figure 2:**
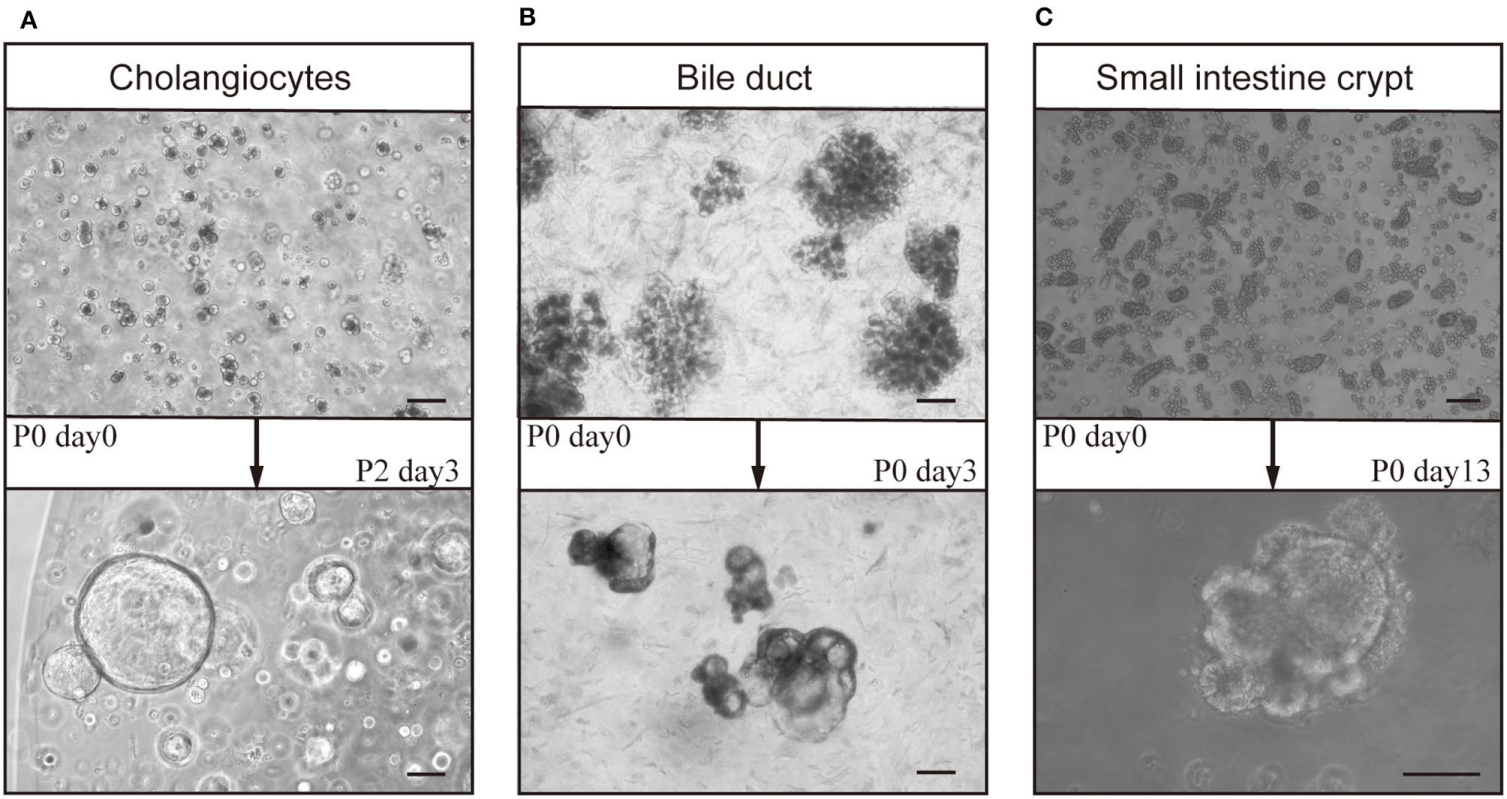
Culture of organoids from bile ducts and small intestines of mice. After gravity settling and multi-step centrifugation, cholangiocytes (derived from bile ducts), bile ducts, and crypts from the small intestine formed organoids after ~14 days in culture. Cholangiocytes (day 0) from **(A)** bile ducts formed cholangiocyte organoids (cho-orgs) (day 3, passage 2) (bar, 100 μm), and those from **(B)** intrahepatic bile ducts (day 0) formed cho-orgs (day 3) (bar, 100 μm). **(C)** Crypts from the small intestine (day 0) (bar, 200 μm) formed small intestinal organoids (in-orgs) (day 13) (bar, 100 μm).

I.i. Collection of bile ducts from mouse liver
  1. To collect liver tissue, anesthetize the mouse and expose the liver. Find the inferior vena cava using a sterile swab, place a sterile cotton ball beside the liver, inject PBS 1× using a 10-mL injector, and cut off the portal vein immediately when the liver swells. By standard surgical procedures, remove the liver as an entire organ and transfer it to a 10-cm Petri dish.
  2. Place the Petri dish on ice and transfer it to a biological safety cabinet. Preheat the digestion solution to 37°C.
  3. Cut the liver tissue into small pieces (<1 mm^3^) using fine scissors. Transfer the small pieces to a 50-mL sterile centrifuge tube, add up to 5 ml precooled washing medium, and pipette it up and down several times using a 10-ml pipette to wash the minced tissue. Repeat the washing procedure.
  4. Transfer the tissue to a new 50-ml sterile centrifuge tube. Add 5 ml pre-warmed digestion medium. Shake the tube at 120–160 rpm and 37°C for ~2 h.
  5. During incubation, check the appearance of bile ducts using a light microscope. Pipette up and down the supernatant in a biological safety cabinet using a 1-ml pipette, and transfer ~200 μl of the solution to a glass slide for observation (Figure 2B, upper row). If no bile ducts are present, return the solution to the shaker. Perform the checking at 20–30 min intervals. Ducts usually appear after 60 min.
  6. When bile ducts appear, transfer the digestion supernatant to a fresh 50-ml centrifuge tube, add the same volume of precooled washing medium, and centrifuge at 80 g for 4 min at 4°C. Discard the supernatant and add 15 mL precooled washing medium; repeat the centrifugation to remove the remaining digestion solution.
  7. Add the precooled washing medium to the pellet and pipette it up and down to mix. Place the 50-mL centrifuge tube upright on ice for 30 min.
  8. Remove the supernatant without disrupting the pellet. Add 5 ml precooled washing medium, transfer the mixture to a fresh 15-ml centrifuge tube, and centrifuge at 60 g for 2 min at 4°C.
  9. Remove the supernatant carefully. Add 1 ml precooled basal medium, transfer the mixture to a 1.5-ml microcentrifuge tube, and centrifuge at 500 rpm for 2 min at 4°C.
  10. The pellet can be directly cultured. If there is a need to collect single bile cells, follow the next procedure.

I.ii. Enrichment of single cholangiocytes
  1. Resuspend bile ducts in 5 ml prewarmed TrypLE solution supplemented with 5 μl DNase I (10 mg/ml). Using narrow 1,000 μl tips, pipette the solution up and down to mix and incubate at 37°C for 2–10 min.
  2. Check the solution every 2 min using a bright-field microscope. Stop the digestion when majority (85–95%) of the mixture consists of single cells by adding the precooled wash medium (Figure 2A, upper row).
  3. Add 10 ml cold washing medium to stop the digestion. Transfer the mixture to a 50-ml centrifuge tube through a 70-μm filter and centrifuge at 300–350 g for 5 min at 4°C. Remove the supernatant and repeat the centrifugation to wash out any remaining TrypLE. Add 1–5 ml washing medium to resuspend the pellet.
  4. Enumerate the cells using a standard cell-counting chamber.

I.iii. Collection of cholangiocytes from gallbladder
  Prewarm the mouse liver digestion medium and TrypLE solution (supplemented with 0.1% [vol/vol) DNase I [10 mg/ml]) to 37°C.
  1. By standard surgical procedures, anesthetize the mouse and expose the liver. Strip the gallbladder using two ophthalmic forceps and transfer it to a 6 mm dish containing precooled mouse liver washing medium.
  2. Cut the gallbladder into small pieces using ophthalmic scissors, transfer the pieces to a 15-ml sterile centrifuge tube using a 1,000 μl Eppendorf pipette, add ~5 ml precooled washing medium, gently pipette the solution up and down, and centrifuge it at 4°C, 200–250 g, for 4 min to remove bile.
  3. Discard the supernatant and add 5 ml mouse liver digestion medium. Shake the tube at 120–180 rpm and 37°C for 1 h.
  4. Add 5 ml precooled washing medium to stop the digestion, centrifuge at 4°C and 200–250 g for 4 min and discard the supernatant.
  5. Resuspend the gallbladder tissue in 1 ml prewarmed TrypLE solution and pipette it up and down 30 times to isolate single cholangiocytes.
  6. Incubate the tube for 2–4 min in a 37°C culture bath and pipette it up and down 30 times.
  7. Add t~5 ml precooled wash medium and filter the mixture through a 70 μm mesh into a 50-ml centrifuge tube using a 1,000 μl Eppendorf pipette. Pipette the fluid into a 15-ml centrifuge tube, and centrifuge at 4°C and 300–350 g for 4 min.
  8. Discard the supernatant and enumerate the cells. The plates are ready for Matrigel embedding and 3D organoid culture.

I.iv. Collection of crypts from mouse small intestine
  1. Anesthetize the mouse; by standard surgical procedures, expose and remove the intestine using ophthalmic forceps. Cut the optional intestine segment 1 cm above the end of the ileum and 2–3 cm below the stomach, and transferred them to a 10 cm dishes containing PBS 1×.
  2. Put the 10-cm dish on ice. Squeeze out the intestinal contents using forceps and cut the tissue longitudinally. Use a blunt instrument (e.g., the curved part of curved dissecting forceps) to scrape the intestinal villi, wash twice or thrice and cut the tissue into 1–2 cm pieces.
  3. Transfer the pieces to a 50 ml centrifuge tube and add 20 ml mouse intestine digestion medium. Put the tube on a shaker at 120–160 rpm and 4°C and incubate for about 30 min.
  4. Carefully remove the supernatant and resuspend the tissue in a new 50 ml centrifuge tube, add ~25 mL PBS 1×, and vigorously pipette the solution up and down 30–50 times to isolate crypts from the tissue.
  5. Filter the supernatant through 100- and 70-μm meshes into a new 50 ml centrifuge tube.
  6. Allow the tube to stand for 8 min and discard the supernatant or transfer them to a new tube for another 8 min-standing circulation. Gather the plates together and check the proportion of the crypts. Add 1–5 ml PBS 1× to the white sediment and pipette it up and down gently. Observe the mixture under a light microscope.
  7. If the proportion of single cells is too high, add ~25ml precooled PBS 1×, and repeat the 8-min standing circulation procedure in step (D-6) to collect a higher proportion of crypts.
  8. Count the crypts using a counting board.

II. Seeding and culture: cholangiocytes/bile ducts and crypts
  1. Prewarm 24-well (or 48-well) culture plates at 37°C for at least 30 min. Pre-dissolve Matrigel and keep it on ice.
  2. Resuspend an appropriate number of cholangiocytes, duct structures, or crypts (e.g., 5,000 cells or 250 duct/crypt structures per well of a 24-well plate) in Matrigel for seeding. For example, use a volume of 40 μl per 24-well plate or 20 μl per 48-well plate.
  3. Put Matrigel in the plates and mix gently. Add a droplet of the mixture (basal matrix and cultures) to the center of each well to prevent the droplet from touching the edges. Incubate for 15–20 min at 37°C until Matrigel solidifies.
  4. Add the appropriate medium to each well (500 μl per well for a 24-well plate or 250 μl per well for a 48-well plate) (liver isolation medium for cho-org culture, intestine expansion medium for in-org culture).
  5. Incubate the plates at 37°C in a 5% CO_2_ atmosphere. For cho-orgs, after 3 days, change the isolation medium with the expansion medium and incubate for ~14 days. For in-orgs, retain the intestine expansion medium. Change the medium every 3–4 days. Organoids will start to develop on days 3–5.

III. Organoid passage
  1. Prewarm culture plates for 1 h–overnight. Place Matrigel on ice and thaw before use. Prewarm the TrypLE Express solution (~2 ml per tube) in a water bath at 37°C.
  2. To disrupt the basal matrix, add 500–1,000 μl precooled basal medium (for cho-orgs) or PBS 1× (in-orgs), and pipette the mixture up and down using a 1,000 μL pipette. Transfer the organoid suspension (three wells for 24-well culture plates or six wells for 48-well culture plates) to a 15 ml centrifuge tube, add ~10 ml precooled basal medium (cho-orgs) or PBS 1× (in-orgs) to the top, and mix by pipetting vigorously 5–10 times to wash away Matrigel.
  3. Centrifuge the tube at 200–250 g for 5 min at 4°C.
  4. Discard the supernatant, leaving 100 μl mixture.
  5. Add the prewarmed TrypLE Express solution and mix by vigorously pipetting up and down using a 1,000 μl pipette. Transfer the tube to a water bath at 37°C for 1–4 min, checking every 2 min under a light microscope. When majority (85–95%) of the material is single cells, add ~10 ml precooled basal medium to stop digestion.
  6. Centrifuge the tube at 300–350 g for 4 min at 4°C, and carefully aspirate the supernatant.
  7. Organoids can be mechanically dissociated and split at a 1:3–1:6 ratio. Resuspend the cells in Matrigel (40–50 μl per well for 24-well plates or 20–25 μl per well for 48-well plates). Pipette the mixture up and down gently to resuspend the cells. Add a droplet of the mixture to the center of each well. Incubate for 15–20 min to allow for Matrigel polymerization.
  8. Overlay the cultures with the expansion medium (500 μl per well for 24-well plates or 250 μl per well for 48-well plates).
  9. Replace the medium every 2–3 days.

IV. Harmonizing the growth rates of organoids
Organoids developed from different primary cells grew at different rates, for some experimental needs, organoids in different growing state are hard to perform experiments. To resolve this problem, we purified cho-orgs with similar growth rates using the procedure below, which can be repeated once to thrice according to growth situation. In addition, intestinal organoids can also use the following method to uniform growth rate.
1) When cho-orgs have budded for 2–3 days (5–7 days after seeding), check their growth state daily under a light microscope.
2) Before cells aggregate in the larger cho-orgs, prepare to selectively passage then, as mentioned in III-1, prewarm the culture plates and TrypLE Express solution, and thaw the Matrigel before use.
3) Move the plates to a sterile environment. Under a light microscope, transfer the larger cho-orgs to the precooled basal medium using a 10-μl pipette.
4) Subsequent steps are the same as for organoids passage; please follow steps III-3–8.

V. Cryopreservation and thawing of organoids
  V.i. Freezing organoids
    1. At least one confluent well (24-well plate) or two confluent wells (48-well plates) of organoids are needed per cryovial tube.
    2. Proceed as in steps (III-1–5) to digest the organoids into single cells and resuspend the cells in 500 μl precooled freezing medium per well (24-well plates) or two wells (48-well plates). Transfer the mixture to cryovials (500 μl each), and immediately place them on ice. Transfer the cryovials at −80°C and then to liquid nitrogen after 24 h. The organoids can be stored for years.
  V.ii. Thawing of organoids
    1. Prewarm a 15 ml tube with 10 ml basal medium (for cho-orgs or in-orgs) at 37°C. Prewarm a 24- or 48-well plate according to need.
    2. Incubate the cryovial in a 37°C water bath and remove when the frozen cell mass is almost completely thawed. Transfer the thawed cell aggregates to the prewarmed basal medium and pipette it up and down gently.
    3. Centrifuge the tube at 250–300 g for 4 min at 4°C.
    4. Remove the supernatant without disturbing the pellet.
    5. Proceed as in steps (II-1–3) to seed the cells in Matrigel and add the appropriate expansion medium (500 μl per well for 24-well plates or 250 μl per well for 48-well plates) to each well.
    6. Replace the medium every 2–3 days.

VI. Analysis of organoids
To characterize the organoids, use option (VI.i) for immunofluorescence analysis, (VI.ii) for immunohistochemical staining, and (VI.iii–iv) for isolation of DNA (iii) or RNA (iv),

VI.i. Immunofluorescence staining
  1. To collect the organoids completely, remove the expansion medium, and add 500–1,000 μl precooled Cell Recovery Solution to each well.
  2. Gently shake the plate horizontally at 4°C for 30–60 min to disrupt Matrigel.
  3. Cut off the top of 1,000 μl tips. Blow the tips twice in precooled 1% (v/v) PBS-BSA, and wash 15 ml centrifuge tubes using 1% (v/v) PBS-BSA to precoat the tips and tubes.
  4. Transfer the mixture to the precoated tubes using the precoated tips, add PBS 1× to ~10 ml and mix gently. Centrifuge at 70 g and 4°C for 4 min, and carefully remove the supernatant.
  5. Repeat the PBS 1× wash steps once or twice to wash out Matrigel completely.
  6. Fix the organoids by resuspending in 4% (w/v) paraformaldehyde at 4°C for 45 min and mix once or twice.
  7. Add ~10 ml precooled 0.1% (vol /vol) PBT, mix, and centrifuge at 70 g and 4°C for 4 min.
  8. Remove the supernatant and resuspend the organoids in the precooled washing buffer. Transfer the mixture to a 24-well plate (>200 μl per well) and incubate at 4°C for 15 min.
  9. When the organoids sink to the bottom, tilt the culture plate at 45° and remove the washing buffer to leave about a 200-μl liquid.
  10. Add a two-fold concentration of primary antibody (diluted in 0.5% PBS-BSA) (200 μL) to each well. Incubate overnight at 60 rpm and 4°C.
  11. Add 1 ml washing buffer to each well and pipette gently.
  12. When the organoids sink to the bottom (3 min), remove 1 ml washing buffer, add 1 mL washing buffer, and incubate for 30 min.
  13. Repeat step (11) at least twice.
  14. When the organoids sink to the bottom, remove the washing buffer to leave a 200 μl liquid.
  15. Add a two-fold concentration of secondary antibody (diluted in 0.5% PBS-BSA) (200 μl) to each well. Incubate overnight at 60 rpm and 4°C.
  16. Repeat 10–13. Transfer the organoids to 1.5 ml EP tubes and centrifuge at 70 g and 4°C for 4 min. The organoids can be stored in the washing buffer at 4°C for 2 days.
  17. Remove the washing buffer without touching the plates.
  18. Add the F-G clearing solution (>50 μl, RT) to the EP tube using top-cut 200-μl tips.
  19. Add an appropriate volume of DAPI and incubate for 20 min at room temperature. The organoids can be stored in the F-G clearing solution for 1 week at 4°C or 6 months at −20°C.
  20. Transfer the organoids to a 24-well plate using 200-μl top-cut tips.
  21. The organoids can be subjected to immunofluorescence imaging.

VI.ii. Immunohistochemical staining of organoids
  1. To obtain the organoids completely, perform steps VI-C 1–6.
  2. Remove 4% (w/v) paraformaldehyde, add ~10 ml precooled PBS 1×, and centrifuge at 70 g and 4°C for 4 min.
  3. Repeat the washing step once or twice.
  4. Remove the supernatant and add ~10 ml 70% alcohol. The organoids can be stored at 4°C in 70% alcohol for 1 week.
  5. Adjust the water bath to 65°C and prewarm Histowax (~3 ml per sample), plastic straws, soft EP tubes, and a metal mold. Be careful to keep the materials dry.
  6. Place a centrifuge tube on ice upright until the organoids sink to the bottom. Remove the supernatant, resuspend the organoids in 0.5% (w/v) eosin solution to dehydrate, and stain for >30 min.
  7. When the organoids sink to the bottom, discard the supernatant and resuspend in 100% alcohol to wash for at least 30 min. Repeat these wash steps thrice.
  8. Resuspend the organoids in dimethylbenzene and wash thrice for ~30 min each.
  9. Remove the dimethylbenzene.
  10. Place the centrifuge tube containing the organoids mentioned above in a 65°C water bath, absorb appropriate volume of prewarmed liquid Histowax (≤500 μL) to the organoids using the prewarmed plastic straws, quickly pipette twice or thrice, and transfer the mixture to the prewarmed soft EP tubes.
  11. Incubate the soft EP tubes at 65°C overnight.
  12. Transfer the soft EP tubes to RT or a cold area to solidify Histowax.
  13. Peel off the tube carefully and cut the Histowax to a smaller size suitable for embedding. This step concentrates the organoids, facilitating their staining and visualization.
  14. Re-embed the small wax block to a new Histowaxin metal mold.
  15. Cool the mold and take out Histowax, which can be stored at RT for years.
  16. Cut Histowax into pieces for further staining.

VI.iii. Isolation of DNA
  1. Perform the procedure in main text III 2–3 to remove Matrigel.
  2. Discard the supernatant, add ~1 ml precooled PBS 1×, and pipette the mixture up and down thrice. Transfer the mixture to 1.5 ml EP, and centrifuge at 250 g and 4°C for 4 min.
  3. Resuspend the organoids in PBS 1×, transfer them to a 1.5 ml EP tube, and centrifuge at 300 g and 4°C for 4 min.
  4. Discard the supernatant and add 30 μl Direct-PCR solution and 0.5 μl proteinase K per sample.
  5. Incubate at 60°C overnight.
  6. Centrifuge at ≥8,000 g at RT for 10 min.
  7. DNA can be stored at 4°C.
  8. If necessary, purify the DNA using a DNA purification kit.

VI.iv. Isolation of RNA
  1. To collect the organoids, follow steps VI.iii 1–3 to collect cell mass.
  2. Remove the supernatant, add 350 μl RLT to each 1.5 ml microcentrifuge tube, and vortex-mix for 30 s.
  3. To promote cell mass lysis, draw the mixtures at least 5 times without RNase using a sterile syringe fitted with a no. 20 needle.
  4. The mixtures can be stored at −80°C for 1 month.
  5. RNA can be extracted following the RNeasy Mini Kit (Qiagen, Hilden, Germany) protocol guides.

### Troubleshooting

Troubleshooting advice can be found in Table 1.

**Table 1:** Troubleshooting.

**Step**	**Problem**	**Possible reason**	**Solution**
I.i-5	Low yield after digestion	Over- or under-digestion of liver tissue	Check the duct cells every 20 min during digestion, (Figure 2A upper row)
I.i-7 I.ii-2	Large amount of cell debris	Over-digestion of the liver tissue or the gravity sinking time was too long	When large flakes of ducts appear, stop the digestion; shorten the gravity sinking time
II-2	Air bubbles in the Matrigel	The Matrigel was pipetted too quickly	Pipetted slowly when seeding; if bubbles are formed, centrifuged tube at 4°C to push the bubbles to the top of the mixture
II-3	Matrigel solidified in the tube or the tip	Over-temperature of Matrigel when seeding	Keep the Matrigel mixture on ice and pre-cold the tips; perform the seeding procedure as soon as possible
III-5	Low formation rate of passaged organoids	Over-digestion of organoids or mechanical separation too fierce.	Check the single cells under a microscope every 2 min; slow down the pipetting speed.
III-7	More cell clumps after digestion	Not enough digestion time or too little mechanical blowing	Increase the digestion time; increase the number of mechanical blowing (10-30 times) before neutralizing the digestion
VI.ii-10	Wax blocks touch softly	Not enough incubation of wax blocks in cold area or too much vapor mixed in the wax.	Prolong the incubation time of wax in cold area and keep care of the vapor
VI.ii-13	Wax blocks crack easily	The wax block has not set completely; peel off the soft EP tube too fiercely.	Prolong the incubation time of wax in cold area; Cut the soft EP tube by fine scissor before peeling off the tube
VI.iv-1	Cell clumps tend to stick to the wall of the tube or on the tips	The Matrigel is not completely removed	Before washing with PBS 1×, washing the Matrigel-cell mixture by medium 1-2 more times; and then avoid the 1000 μL tips going too deep under the liquid surface when washing with PBS 1×

### Step Times

Step I. i–ii: collection of mouse liver bile-duct cells: 4 h.
Step I. iii: collection of mouse gallbladder cholangiocytes: 2 h.
Step I. iv: isolation of mouse intestinal crypts: 2 h.
Step II: seeding of mouse cholangiocytes or crypts: 30 min.
Step III: passaging of mouse organoids: 30–45 min.
Step IV: harmonizing of organoid growth rate: 40–60 min.
Step V: cryopreservation and thawing of mouse organoids: 30 min each.
Step VI: analysis of organoids: 2 days for isolation of DNA, 40–60 min for isolation of RNA, 3 days for immunofluorescence staining, and 2 days for immunohistochemical staining.

## Results

### Establishment of Organoid Cultures

The bile ducts, cholangiocytes, and intestine crypts embedded in Matrigel developed into 3D structures (Figure 2). The cho-orgs from cholangiocytes were single cystic spheres, while the primary cho-orgs from bile ducts comprised multi-cystic sphere-like organoids but can be separated after passaging and grow into single cystic spheres, as in the case of cho-orgs derived from single cholangiocytes. Besides, the In-orgs were bud-shaped and had aggregated cell clumps in the organoids.

### Growth of Organoids

**Figure 3:**
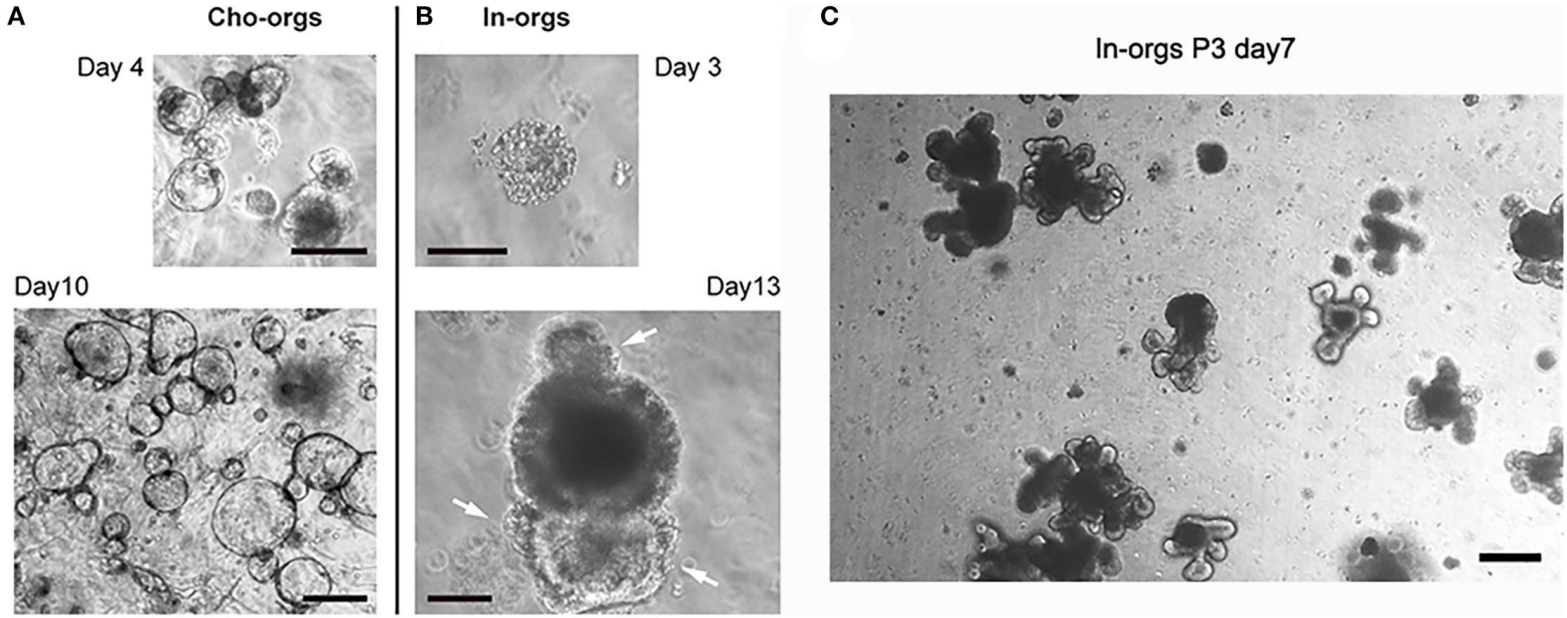
Development of cho-orgs and in-orgs. Cholangiocytes (bile ducts) and crypts from the small intestine embedded in Matrigel developed into cyst-like structures. **(A,B)** Cells congregated in the cysts of in-orgs (day 3) and were outside the cho-orgs (day 4); after culturing for 10–13 days, the cho-orgs (day 10) and in-orgs (day 13) increased in size, and the in-orgs developed three buds (white arrow) to form a mature structure (bar, 100 μm). **(C)** Mature in-orgs after culturing for 7 days after passage, they were bud-shaped and had aggregated cell clumps in the organoids (bar, 100 μm).

After seeding for 2–4 days, primary stem cells from the cholangiocytes or small intestine started to develop into cyst-like structures (Figure 3A). After 10–14 days, the organoids increased in size and matured (Figure 3B) and could be passaged at a 1:3–1:4 ratio for further expansion (Figures 2A, 3C).

### Characteristics of Cho-Orgs and In-orgs

**Figure 4:**
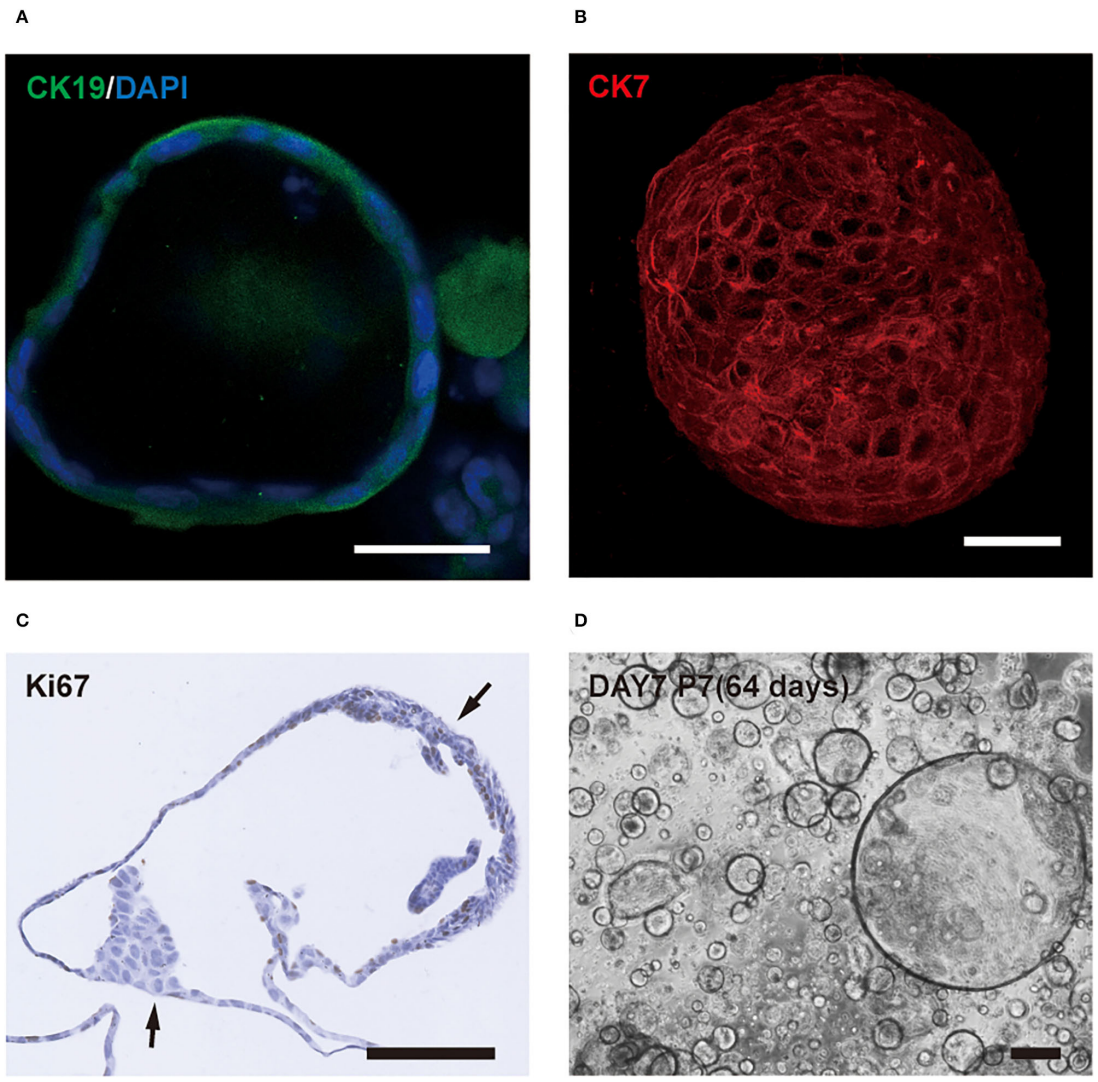
Characteristics of cholangiocytes-organoids (cho-orgs). **(A)** Immunofluorescence staining for the cholangiocyte marker CK19 (green) (bar, 100 μm). **(B)** Immunofluorescence staining for CK7 (red) in a 3D structure (bar, 100 μm). **(C)** The cho-orgs consisted of more than one cell layer and express the proliferation marker Ki67 (bar, 100 μm). **(D)** The cho-orgs from intrahepatic bile ducts were cultured for 7 days after seeding and passaged seven times for a total culture duration of >64 days (bar, 200 μm).

Cho-orgs expressed the cholangiocytes markers Krt19 (Figure 4A) and Krt7 (Figure 4B), showed high proliferative activity, and consisted of more than one cell layer (Figure 4C).

We found a phenomenon that the cho-orgs from the intrahepatic bile ducts grew slower than the cho-orgs from gallbladders. Formation of mature organoids, about 200 nm in size, took only about 5 days for gallbladder-derived bile duct cells compared to the 7-14 days for primary intrahepatic bile duct cells. However, the latter could be passaged for a longer period (Figure 4D).

**Figure 5:**
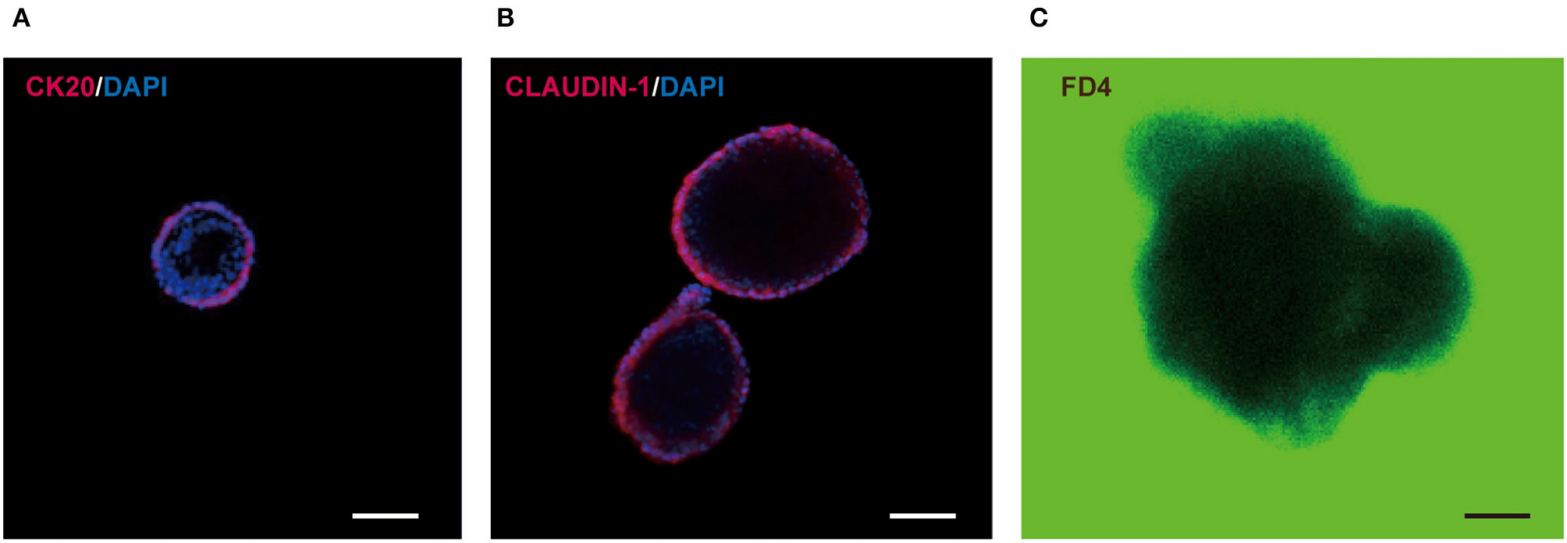
Characteristics of small intestinal organoids (in-orgs). **(A)** Immunofluorescence staining for the enterocyte cell marker CK20 (red) (bar, 100 μm). **(B)** Immunofluorescence staining for the complete tight junction protein CLAUDIN-1 (red) (bar, 100 μm). **(C)** Fluorescent dye FITC-dextran-4KD (FD4) absorbing results, observed with a laser confocal Microscope. The in-orgs are on a green background (bar, 100 μm).

Besides, the in-orgs expressed the enterocyte cell marker CK20 before they matured (Figure 5A), and the cell connections were stable as shown in the complete tight junction protein CLAUDIN-1 staining results (Figure 5B). In addition, the intestinal permeability of the in-orgs was proved to be normal, and the fluorescent dye did not enter the cavity of the organoid, as identified by FITC-dextran-4KD (FD4) staining analysis (Figure 5C).

It is worth mentioning that the digestion time of the organoids in TrypLE Express solution is crucial for the viability of the next generation of organoids during passaging. On the one hand, prolonged incubation time leads to over-digestion and lower cell viability; on the other hand, insufficient digestion can lead to decrease in the number of organoids. Therefore, the digestion situation of organoids should be monitored under a light microscope. The optimum time of digestion can be determined when 80–90% of cells are single cells.

### Stable Growth of Cho-Orgs After Cryopreservation and Recovery

**Figure 6:**
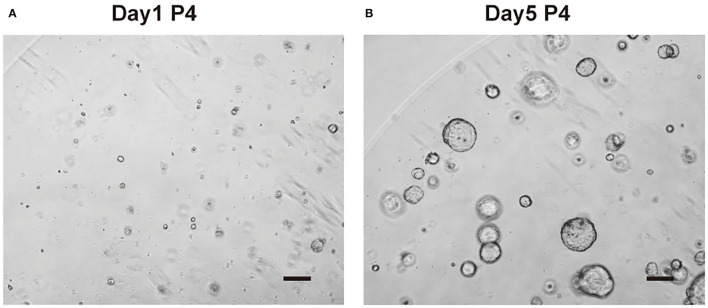
Organoid culture after thawing. **(A)** Cholangiocytes-organoids (cho-orgs) were cultured for 1 day after thawing (bar, 200 μm) (P4, passage 4). **(B)** Cho-orgs are cultured for 5 days after thawing (bar, 200 μm).

The organoids grew stably after thawing, as observed by light microscopy (Figure 6). After stable culture, homogenization of growth rate manipulation, the organoids were ready for RNA/DNA isolation, immunochemical staining, cryopreservation, or further experiments.

## Conclusions

Our protocols have high repeatability if tissue dissociation steps are performed correctly, and the composition of medium is precise. Besides, we simplify the cell collection procedure by gravity settling and multistep centrifugation to collect cholangiocytes/bile duct and intestine crypts from mouse liver cholangiocytes or small intestine, although some unwanted cells may also be collected during the process, which will disappear gradually after 1–2 passages of the organoids. In addition, when experiments are operated properly, the possibility of exposing stem cells to mechanical damage or chemical injury will be avoided. After passaging, cryopreservation, and thawing, the organoids are in a stable growth state and suitable for mechanistic analysis, preclinical research, drug screening or further experiments.

## Data Availability Statement

The original contributions presented in the study are included in the article/Supplementary Material, further inquiries can be directed to the corresponding author/s.

## Ethics Statement

The animal study was reviewed and approved by the First Affiliated Hospital, College of Medicine, Zhejiang University (reference number 2020-1088).

## Author Contributions

WC and HC contributed to the design of the study, method optimization, and writing of the manuscript. WC, QY, and RW contributed to organoid culture and experiments. WC, BF, JC, YX, and JY contributed to method optimization. WC and QY contributed to the production of Figures. LL conducted study supervision. All authors have read and approved the final version of the manuscript.

## Funding

This study was supported by grants for the Stem Cell and Translational Research from National Key Research and Development Program of China (No. 2020YFA0113003) and National Natural Science Foundation of China (No. 81971756).

## Conflict of Interest

The authors declare that the research was conducted in the absence of any commercial or financial relationships that could be construed as a potential conflict of interest.

## Publisher's Note

All claims expressed in this article are solely those of the authors and do not necessarily represent those of their affiliated organizations, or those of the publisher, the editors and the reviewers. Any product that may be evaluated in this article, or claim that may be made by its manufacturer, is not guaranteed or endorsed by the publisher.

## Abbreviations

3D: three dimensional
PSC: primary sclerosing cholangitis
IBD: inflammatory bowel diseases
EGF: epidermal growth factor
FGH: fibroblast growth factor
HGF: hepatocyte growth factor
ROCK: Rhokinase
IF: immunofluorescence
IHC: immunohistochemistry.
