# Supplementary material for: Highly Efficient Methods to Culture Mouse Cholangiocytes and Small Intestine Organoids
Source: Front Microbiol. 2022 May 20;13:907901. doi: 10.3389/fmicb.2022.907901 (PMC9164252; doi:10.3389/fmicb.2022.907901)
Supplement: Supplementary file 1 [file Table_1.docx]

**Supplementary Material**

**Highly efficient methods to culture mouse liver and small intestine organoids**

Wenyi Chen^1,2^, Qigu Yao^1,2^, Ruo Wang^1,2^, Bing Fen^1,2^, JunYao Chen^1,2^, Yanping Xu^1,2^, Jiong Yu^1,2^, Lanjuan Li^1,2^, Hongcui Cao†^1,2,3^

1 State Key Laboratory for the Diagnosis and Treatment of Infectious Diseases, Collaborative Innovation Center for Diagnosis and Treatment of Infectious Diseases, The First Affiliated Hospital, Zhejiang University School of Medicine, 79 Qingchun Rd., Hangzhou City 310003, China

2 National Clinical Research Center for Infectious Diseases, 79 Qingchun Rd., Hangzhou City 310003, China

3 Zhejiang Provincial Key Laboratory for Diagnosis and Treatment of Aging and Physic-chemical Injury Diseases, 79 Qingchun Rd, Hangzhou City 310003, China.

**†Corresponding author:**

Hongcui Cao, M.D.

State Key Laboratory for the Diagnosis and Treatment of Infectious Diseases, The First Affiliated Hospital, Zhejiang University School of Medicine, 79 Qingchun Rd., Hangzhou City 310003, China. Tel: 86-571-87236451; Fax: 86-571-87236459

E-mail: [hccao@zju.edu.cn](mailto:hccao@zju.edu.cn)

**MATERIALS**

**Table S1.** **Reagents of organoids culture and analysis.**

| Reagents and Materials | | |
| --- | --- | --- |
| PBS 1× | RuiSenBiotech | - |
| FBS | Gibico | 10099-141C |
| 4% (wt/vol) paraformaldehyde | Absin | abs9179-500mL |
| RNeasy Mini Kit | Qiagen | 74104 |
| DNeasy Blood & Tissue kit | Qiagen | 69506 |
| BSA | Sangon Biotech | A0332 |
| Histowax (58-60 ℃) | Hualingpai | A-11001 |
