# Supplementary material for: Highly Efficient Methods to Culture Mouse Cholangiocytes and Small Intestine Organoids
Source: Front Microbiol. 2022 May 20;13:907901. doi: 10.3389/fmicb.2022.907901 (PMC9164252; doi:10.3389/fmicb.2022.907901)
Supplement: Supplementary file 2 [file Table_2.docx]

**Supplementary Material**

**Highly efficient methods to culture mouse liver and small intestine organoids**

Wenyi Chen^1,2^, Qigu Yao^1,2^, Ruo Wang^1,2^, Bing Fen^1,2^, JunYao Chen^1,2^, Yanping Xu^1,2^, Jiong Yu^1,2^, Lanjuan Li^1,2^, Hongcui Cao†^1,2,3^

1 State Key Laboratory for the Diagnosis and Treatment of Infectious Diseases, Collaborative Innovation Center for Diagnosis and Treatment of Infectious Diseases, The First Affiliated Hospital, Zhejiang University School of Medicine, 79 Qingchun Rd., Hangzhou City 310003, China

2 National Clinical Research Center for Infectious Diseases, 79 Qingchun Rd., Hangzhou City 310003, China

3 Zhejiang Provincial Key Laboratory for Diagnosis and Treatment of Aging and Physic-chemical Injury Diseases, 79 Qingchun Rd, Hangzhou City 310003, China.

**†Corresponding author:**

Hongcui Cao, M.D.

State Key Laboratory for the Diagnosis and Treatment of Infectious Diseases, The First Affiliated Hospital, Zhejiang University School of Medicine, 79 Qingchun Rd., Hangzhou City 310003, China. Tel: 86-571-87236451; Fax: 86-571-87236459

E-mail: [hccao@zju.edu.cn](mailto:hccao@zju.edu.cn)

**MATERIALS**

**Table S2.** **General Instruments of organoids culture and analysis.**

| General Instruments | | |
| --- | --- | --- |
| Biological safety cabinet | Baker company | MODEL: SG403A-HE-INT |
| Cell culture incubator | Thermo | STERI-CYCLE i160 |
| Bright-field microscope | Nikon | TS100 |
| High-speed centrifuge | Eppendorf | 5810 R |
| Water bath | Thermo Fisher Scientific | ISOTEMP 215 |
| Temperature-controlled shaker | SZ | ZHSY-50S |
| Low temperature shaker | Thermo | 888-213-1790 |
| Fine scissors | Suzhou Medical Equipment | MR-S231 |
| Two fine forceps | Shanghai Medical instrument | 180801 |
| Glass Pasteur pipette | Thermo | 10006021 |
| Centrifuge tubes | Thermo, Corning | 339652, CLS430791 |
| 1.5 mL Microcentrifuge tubes | Corning | MCT-150C |
| Soft Microcentrifuge tubes | Corning | MCT-200-C |
| 10-mm Petri dish | Corning | 353003 |
| 24-Well adherent culture plate | Corning | 353047 |
| Disposable sterilized syringe | Nantong Tiansheng Experimental Equipment | LTD, 10 mL and 1 mL |
| Sterile swabs | Huabao | Sterile type 80 mm |
| Ice box | Esky | 1257470 |
| 5 mL Flat-bottom glass tubes | Thermo Fisher Scientific | 11563542 |
| Tissue cassette | VWR | 720-0228 |
| Glass slides | Nantong Tiansheng Experimental Equipment | TS-PNSO23 |
| Coverslips | CITOTEST | 10212424C |
