# Supplementary material for: Highly Efficient Methods to Culture Mouse Cholangiocytes and Small Intestine Organoids
Source: Front Microbiol. 2022 May 20;13:907901. doi: 10.3389/fmicb.2022.907901 (PMC9164252; doi:10.3389/fmicb.2022.907901)
Supplement: Supplementary file 3 [file Table_3.docx]

**Supplementary Material**

**Highly efficient methods to culture mouse liver and small intestine organoids**

Wenyi Chen^1,2^, Qigu Yao^1,2^, Ruo Wang^1,2^, Bing Fen^1,2^, JunYao Chen^1,2^, Yanping Xu^1,2^, Jiong Yu^1,2^, Lanjuan Li^1,2^, Hongcui Cao†^1,2,3^

1 State Key Laboratory for the Diagnosis and Treatment of Infectious Diseases, Collaborative Innovation Center for Diagnosis and Treatment of Infectious Diseases, The First Affiliated Hospital, Zhejiang University School of Medicine, 79 Qingchun Rd., Hangzhou City 310003, China

2 National Clinical Research Center for Infectious Diseases, 79 Qingchun Rd., Hangzhou City 310003, China

3 Zhejiang Provincial Key Laboratory for Diagnosis and Treatment of Aging and Physic-chemical Injury Diseases, 79 Qingchun Rd, Hangzhou City 310003, China.

**†Corresponding author:**

Hongcui Cao, M.D.

State Key Laboratory for the Diagnosis and Treatment of Infectious Diseases, The First Affiliated Hospital, Zhejiang University School of Medicine, 79 Qingchun Rd., Hangzhou City 310003, China. Tel: 86-571-87236451; Fax: 86-571-87236459

E-mail: [hccao@zju.edu.cn](mailto:hccao@zju.edu.cn)

**MATERIALS**

**Table S3. Antibodies of organoids analysis.**

| Antibodies | | |
| --- | --- | --- |
| Goat Anti-Rabbit IgG H&L (HRP) | Abcam | ab6721 |
| Rabbit Anti-Mouse IgG H&L (HRP) | Abcam | ab6728 |
| Recombinant Anti-Cytokeratin 7 antibody [EPR17078] | Abcam | ab181598 |
| Anti-Ki67 antibody [SP6] | Abcam | ab16667 |
| Anti-Cytokeratin 19 antibody [A53-B] | Abcam | ab194399 |
| Goat Anti-Rabbit IgG H&L (Alexa Fluor® 647) | Abcam | ab150083 |
| Goat anti-Mouse IgG (H+L) Cross-Adsorbed Secondary Antibody, Alexa Fluor 488 | Invitrogen | A-11001 |
| DAPI | BD Pharmingen | 564907 |
